# Review of Facial Nerve Anatomy: Trauma to the Temporal Region

**Published:** 2013-07-29

**Authors:** Will Schleicher, Michael Feldman, Jennifer Rhodes

**Affiliations:** Department of Surgery, Division of Plastic Surgery, Virginia Commonwealth University, Richmond

**Keywords:** facial nerve, SMAS, temporoparietal fascia, stylomastoid foramen, temporal branch

**Figure:**
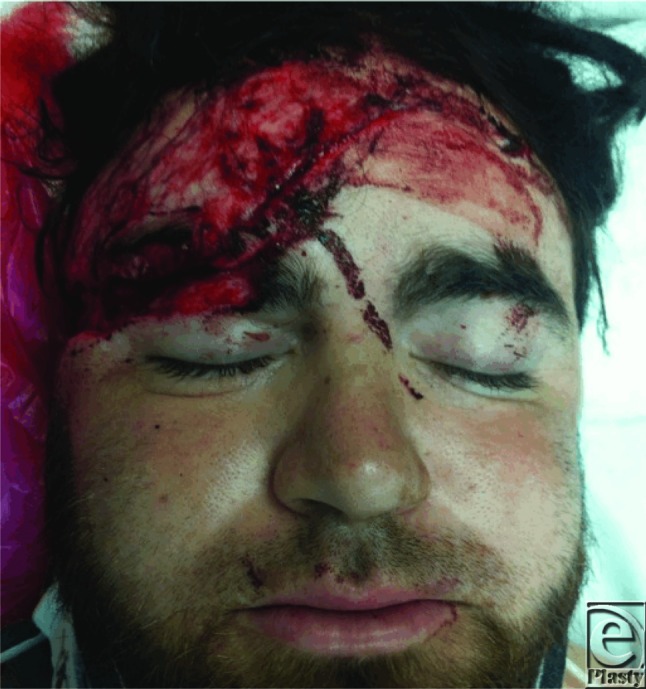


## DESCRIPTION

A 25-year-old man presented as a trauma alert to the emergency department after being involved in a motor vehicle crash. After appropriate ATLS (advanced trauma life support) evaluation, a large bulky head wrap was removed revealing a 25-cm transverse laceration extending from the medial brow traversing the most cephalad portion of the right superior helix, and extending toward occiput.

## QUESTIONS

1. **What is the course of the main trunk of the facial nerve after it exits the cranium?**
2. **Where does the facial nerve divide?**
3. **What are the branches of the facial nerve?**
4. **Through which tissue planes does the facial nerve travel as it crosses the temporal region?**

## DISCUSSION

The patient presented with a 20-cm, irregular, transverse, laceration with significant debris in the wound base. This complex laceration to the right frontal/temporal region provides an excellent opportunity to review the anatomy of the facial nerve. It is not uncommon to encounter an unrestrained occupant of motor vehicle in the trauma setting. Without restraining devices, patients regularly sustain polytrauma including facial fractures and concomitant soft tissue injuries. As plastic surgeons treating these injury patterns, it is vital to understand the anatomic foundations of the face. The basics of bony architecture, vascularity, and innervation are among those necessary concepts to master.

A review of the anatomy in the temporal region is a point to return to in many traumatic and elective surgical interventions due to the complexity of the facial planes enveloping the facial nerve. The facial nerve exits the cranium at the stylomastoid foramen just inferior and posterior to the auricle. It has the longest path within a bony canal of any nerve in the body. Upon its exit of the cranium, the nerve courses within the parotid gland in a superior medial direction. It is within the gland itself, at the pes anserinus, that it separates into the temporozygomatic and cervicofacial divisions. These divisions course superior and inferior, respectively. As the divisions proceed in an anterior direction, they separate into 5 branches: frontal/temporal, zygomatic, buccal, mandibular, and cervical. The temporal region itself presents some of the most challenging anatomic layout due to the numerous facial layers. The course of the temporofrontal branch can be estimated using external landmarks as described by Pitanguy et al. A line starting from a point 0.5 cm below the tragus in the direction of the eyebrow, passing 1.5 cm above the lateral extremity of the eyebrow estimates the path of the nerve in the soft tissue.^2^

The temporal region is unique in its multitude of facial and muscular planes. The nomenclature and unique extension of these planes contribute to the difficult understanding the anatomy of the region. An understanding of these relationships is crucial to knowing the path of the facial nerve. The 3 layers of fascia in the temporal region which have a close relationship to the facial nerve are as follows: the temporoparietal fascia (also called the superficial temporal fascia) and the deep temporal fascia (which is composed of a superficial and deep layer). The temporoparietal fascia is in direct continuity with the galea cephalad, the SMAS (superficial musculo-aponeurotic system) inferiorly, the frontalis anteriorly, and the occipitalis posteriorly.

The temporal branch of the facial nerve courses within these planes at known levels providing a road map for safe dissection.^4^ A review of this anatomy is beneficial to ensure the safe dissection of tissue and avoidance of injury, as well as to establish an idea of injury patterns.

## Figures and Tables

**Figure 1:**
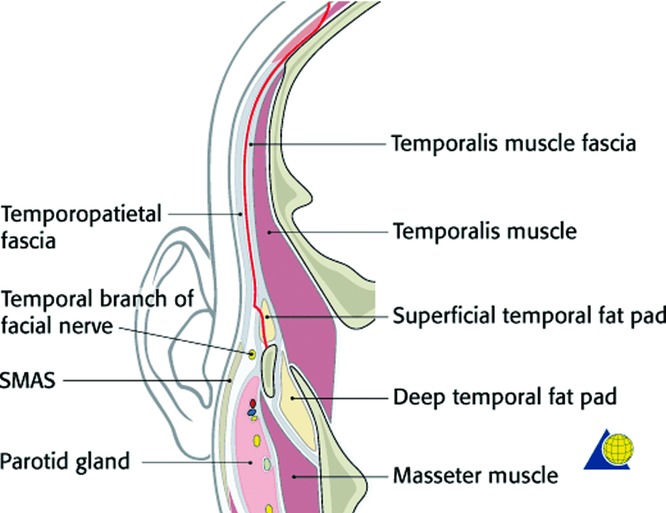
Diagram of the spatial relationship of the facial nerve in the temporal region coursing deep to the SMAS and superficial to the superficial layer of the deep temporal fascia. Copyright by AO Foundation, Switzerland. Source: AO Surgery Reference, www.aosurgery.org.

**Figure 2:**
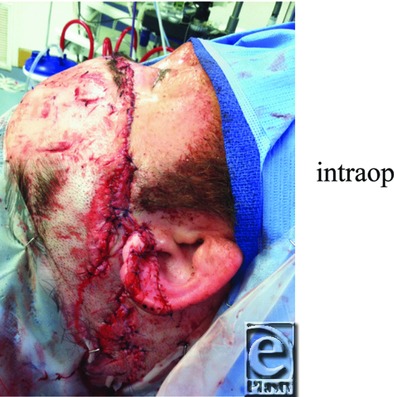
Intraoperative photograph post washout and laceration repair.

**Figure 3:**
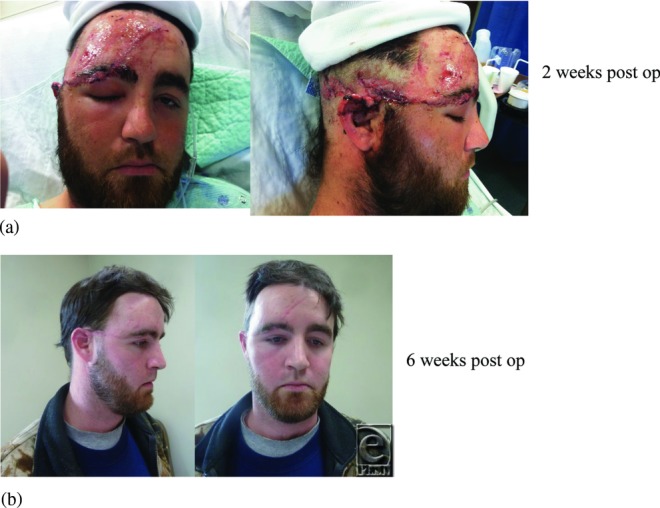
Postoperative photographs at follow-up (a) 2 weeks, (b) 6 weeks.
